# Neurochemical and microstructural alterations in bipolar and depressive disorders: A multimodal magnetic resonance imaging study

**DOI:** 10.3389/fneur.2023.1089067

**Published:** 2023-03-01

**Authors:** Lingmei Kong, Hui Li, Fengfeng Lin, Wenbin Zheng, Haidu Zhang, Renhua Wu

**Affiliations:** ^1^Department of Medical Imaging, The Second Affiliated Hospital of Shantou University Medical College, Shantou, China; ^2^Department of Psychiatry, Shantou University Mental Health Center, Shantou, China

**Keywords:** bipolar disorder, unipolar depression, magnetic resonance spectroscopy, chemical exchange saturation transfer (CEST) for glutamate, diffusion kurtosis imaging

## Abstract

**Aims:**

Depression in bipolar disorder (BD) is often misdiagnosed as unipolar depression (UD), leading to mistreatments and poor clinical outcomes in many bipolar patients. Herein, we report direct comparisons between medication-free patients with BD and those with UD in terms of the microstructure and neurometabolites in eight brain regions.

**Methods:**

A total of 20 patients with BD, 30 with UD patients, and 20 matched healthy controls (HCs) underwent 3.0T magnetic resonance imaging with chemical exchange saturation transfer (CEST) for glutamate (Glu; GluCEST) imaging, multivoxel magnetic resonance spectroscopy, and diffusion kurtosis imaging.

**Results:**

Compared with HCs, patients with UD showed significantly lower levels of multiple metabolites, GluCEST% values, and diffusional kurtosis [mean kurtosis (MK)] values in most brain regions. In contrast, patients with BD presented significantly higher levels of Glu in their bilateral ventral prefrontal white matter (VPFWM), higher choline (Cho)-containing compounds in their left VPFWM and anterior cingulate cortex (ACC), and higher GluCEST% values in their bilateral VPFWM and ACC; moreover, reduced MK in these patients was more prominent in the left VPFWM and left thalamus.

**Conclusion:**

The findings demonstrated that both patients with UD and BD have abnormal microstructure and metabolic alterations, and the changes are not completely consistent in the prefrontal lobe region. Elevated Glu, Cho, and GluCEST% in the ACC and VPFWM of patients with UD and BD may help in differentiating between these two disorders. Our findings support the significance for the microstructural integrity and brain metabolic changes of the prefrontal lobe region in BD and UD.

## 1. Introduction

Bipolar disorder (BD) is a mental disorder characterized by recurrent episodes of elevated mood and depression, and unipolar depression (UD) is characterized by depressed mood, loss of interest, slow thinking, low energy, and a wide range of physical characteristics such as early awakening, heavy sleep at night, loss of appetite, and weight loss. BD is a primary cause of disability among young people that causes cognitive and functional impairment and increased mortality, particularly due to death by suicide (1). Accurate diagnosis of BD is difficult in clinical practice because individuals with BD spend the majority of their time experiencing depression, which is typically the presenting symptom; therefore, many patients with BD are initially diagnosed and treated for UD. As the treatment regimen is different for these two mood disorders, the misdiagnosis, in turn, leads to poor prognosis and increased suicide rates and health care costs. Therefore, correct early diagnosis is of utmost importance.

Because distinguishing BD from UD solely based on clinical clues is difficult, other promising neural markers are being explored using neuroimaging measures, and non-invasive functional magnetic resonance imaging (MRI) has produced promising results (2, 3). Non-invasive *in vivo* measures of brain structure and function derived from MRI have shed light on the underlying brain alterations associated with BD. However, while prior studies have found relative consensus across studies, conflicting results are not uncommon in neuroimaging studies of BD (4). Diffusion kurtosis imaging (DKI) is a newly emerging MRI modality based on the non-Gaussian diffusion of water in biological systems and the index of kurtosis. A scalar index derived from DKI called the mean kurtosis (MK) measures the degree of diffusion restriction and indicates microstructural complexity (5). Indeed, its performance has been satisfactory and superior to that of diffusion tensor imaging (DTI) in identifying microstructural abnormalities in several cerebral pathologies, such as neonatal acute bilirubin encephalopathy (6), microstructural changes in brain regions upon acute alcohol intake (7), congenital sensorineural hearing loss (8), and BD (9). In a study on patients with BD, DKI could detect microstructural brain alterations in patients with BD and major depressive disorder (MDD) (10). However, the conclusions on DKI data have not yet been fully studied, it remains unclear to whether DKI abnormalities can distinguish depressed bipolar disorder and unipolar depression.

*In vivo* multivoxel proton magnetic resonance spectroscopy (MRS) of the human brain allows for non-invasive quantification of neurobiochemical compounds such as acetylaspartate (NAA), myoinositol (MI), choline (Cho)-containing compounds, total creatine (Cr), glutamate (Glu), and glutamine+glutamate (Glx), simultaneously assessing a large number of brain regions. However, some neurometabolites, including Glu, glutamine, Glx, and NAA, are implicated in the neurobiological mechanisms of BD (11). Xu et al. (12) reported a higher ratio of Glx/Cr in the left thalamus of depressed patients with BD and lower ratios of Glx/Cr and Glu/Cr in the posterior cingulate cortex (PCC) of hypomanic patients with BD. In another study, Li et al. (13) found increased Glx levels in the anterior cingulate cortex (ACC) and decreased NAA levels in the parietal cortex and medial prefrontal cortex of patients with BD by applying multivoxel MRS. In a recent meta-analysis, Magnotta et al. (14) reported elevated NAA and Glu concentrations in the cerebellar vermis of patients with type I BD.

These findings indicate that the neurobiological mechanisms of BD remain unclear. A previous study has shown that abnormalities in the glutamatergic system in the brain play a role in the pathophysiology of depression (15), and the involvement of Glu and Glx in the pathophysiology of BD has been explored by MRS (12, 13). However, there are currently no valid imaging biomarkers for the disorder; therefore, determining the role of Glu in BD is of utmost importance. Chemical exchange saturation transfer (CEST) is an important contrast mechanism in molecular MRI. As GluCEST contributes a large part of the asymmetric magnetization transfer (MT) ratio (MTRasym) signal at 3 ppm (16), the MTRasym analysis can be used to quantify alterations in Glu concentrations. Although both MRS and GluCEST can non-invasively reflect Glu changes *in vivo*, GluCEST imaging has multiple advantages over MRS. In the present study, taking advantage of DKI, multivoxel MRS, and GluCEST, which allow simultaneous measurement of microstructures and neurochemicals—especially Glu—in several brain regions within a single slice, we directly compared patients with BD with those with UD in terms of microstructures and neurometabolites in eight brain regions to investigates the differences between depressed bipolar and unipolar disorders; matched healthy controls were included for reference.

## 2. Materials and methods

### 2.1. Participants

Twenty patients with BD and 30 with UD with a history of being medication-free for at least 2 weeks before recruitment were included in this study. Twenty healthy controls (HCs) matched for age and sex were enrolled *via* advertisement. Participants in the BD and UD groups met the DSM-IV criteria for BD and UD based on the Structured Clinical Interview for DSM-IV Patient Edition (SCID-P) carried out by two experienced psychiatrists. All patients with BD were identified as depressed when interviewed by the psychiatrists. The Hamilton Depression Rating Scale (HDRS) was used to assess the severity of depressive symptoms. The exclusion criteria for all participants were current serious medical conditions, a history of head trauma, organic mental disorders and neurological disorders, a history of substance abuse or dependence, and age <18 or >60 years. All procedures were performed in accordance with the ethical standards of the Second Affiliated Shantou Medical University Hospital and institutional review boards. Written informed consent was obtained from all participants before entering the study.

### 2.2. MRI

**Figure 1:**
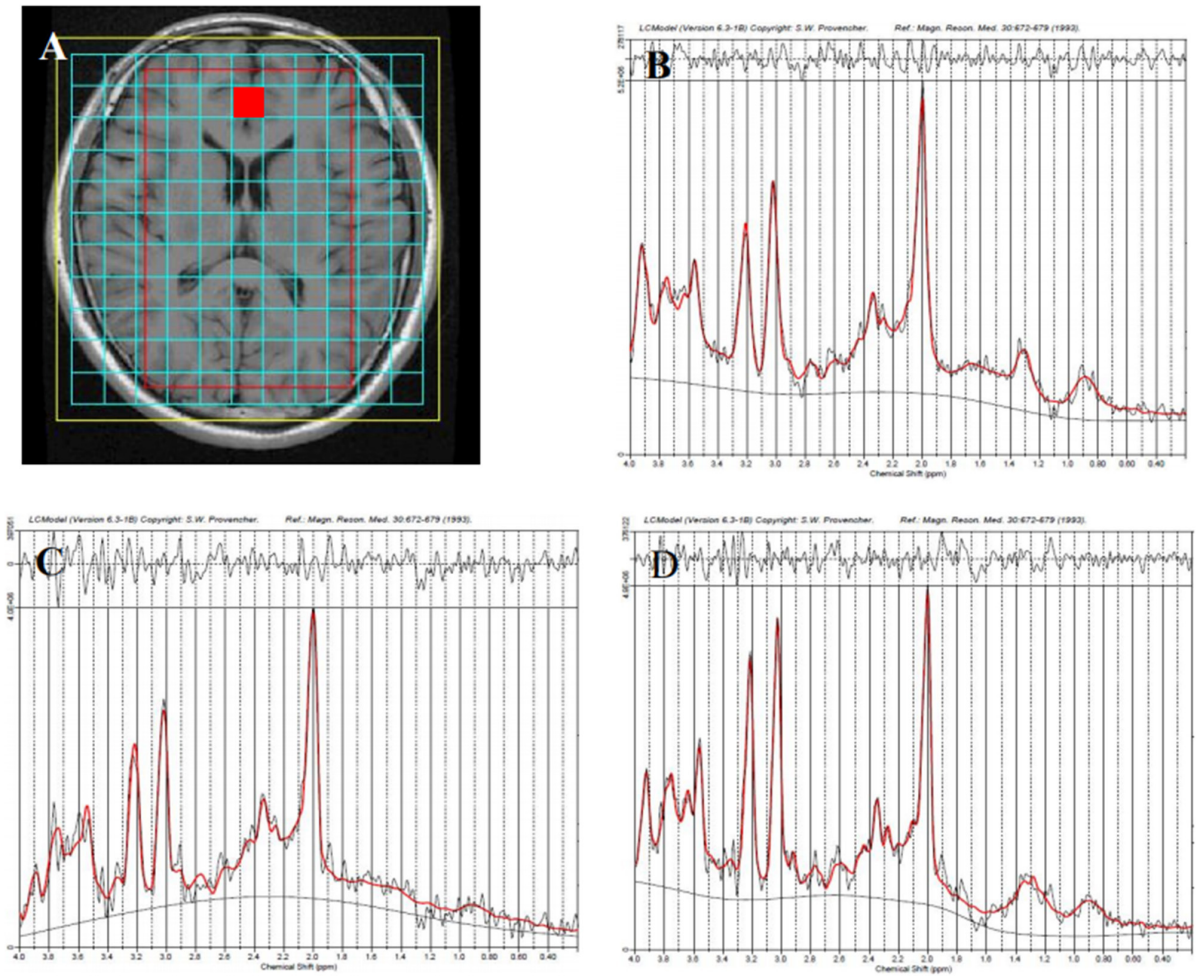
Representative proton magnetic resonance spectroscopy (^1^H-MRS) spectra for the anterior cingulate cortex (red square) in different groups. Region of interest (ROI) is shown in axial T1-weighted imaging (T1WI) **(A)**, the healthy control group **(B)**, the unipolar depression group **(C)**, and the bipolar disorder group **(D)**.

Routine T1 fluid attenuated inversion recovery (T1- FLAIR), Prop T2WI, DKI, MRS, and GluCEST were performed using a 3.0T GE MRI system (Signa, General Electric Medical System, USA) with an eight-channel head coil (GE Medical Systems). T1- FLAIR and prop were performed to confirm the absence of structural or signal abnormalities in the brain. The parameters of these sequences are as follows: T1- FLAIR: repetition time (TR) = 2,000 ms; echo time (TE) = 25 ms, 1 min and 42 s; prop T2WI: TR = 5,000 ms; TE = 104.70 ms 1 min and 35 s. DKI: echo planar imaging was used for DKI acquisition; TR/TE = 6,000/73.4 ms; slice thickness = 3 mm with 1-mm gap; field of view (FOV) = 24 × 24 cm; Freq = 128, Phase = 128; and DKI was applied in 15 encoding diffusion directions at three b values (0, 1,000, and 2,000 s/mm^2^), 4 min and 06 s. MRS was performed using a point-resolved spectroscopy sequence (PRESS) with the following parameters. Single-voxel MRS was performed on the position of the anterior cingulate cortex, all voxel size were equal in size (2 × 2 × 1 cm). TR = 1,500 ms, TE = 30 ms, 3 min 48 s. Multivoxel MRS: all regions of interests (ROIs) were localized at the axial T1WI centrum semiovale level for anatomical localization (Figure 1A). All ROIs were equal in size (8 × 10 × 1 cm^3^), TR = 3,500 ms, TE = 29 ms, FOV = 16 cm × 16 cm, frequency = 12, phase = 12, and NEX = 1, 8 min 38 s. Shimming (line width <20 μm) and water suppression (≥95%) were automatically performed using a variable pulse power and optimized relaxation delay scheme. The water suppression for MRS was performed using CHESS (Chemical shift selective) automatically. If the effect of automatic water suppression is not well, we will adjust the three variables f01, f02 and f03 in the RSP variable, and f03 RSP variable can best optimize the water suppression of MRS generally.

GluCEST: The GluCEST scan was based on an MT-prepared gradient echo (GRE) MRI sequence using Mao (17). The MT saturation pulse was a Fermi pulse with 20-ms width and B_1_ of 1.95 μT, TR = 50 ms, TE = 3.1 ms, FOV = 24 × 24 cm, matrix = 128 × 128, bandwidth = 15.63 kHz, 1 slice, slice thickness = 5 mm. GluCEST imaging was performed on the same brain slice as MRS. Forty-one equidistant frequency offsets from 5 to −5 ppm and S_0_ images were acquired. The average measurement time for CEST in this study is around 25 min. Foam pads were used to reduce head movements. The participants were asked to lay still with their eyes closed during data acquisition.

### 2.3. Data processing

#### 2.3.1. DKI

**Figure 2:**
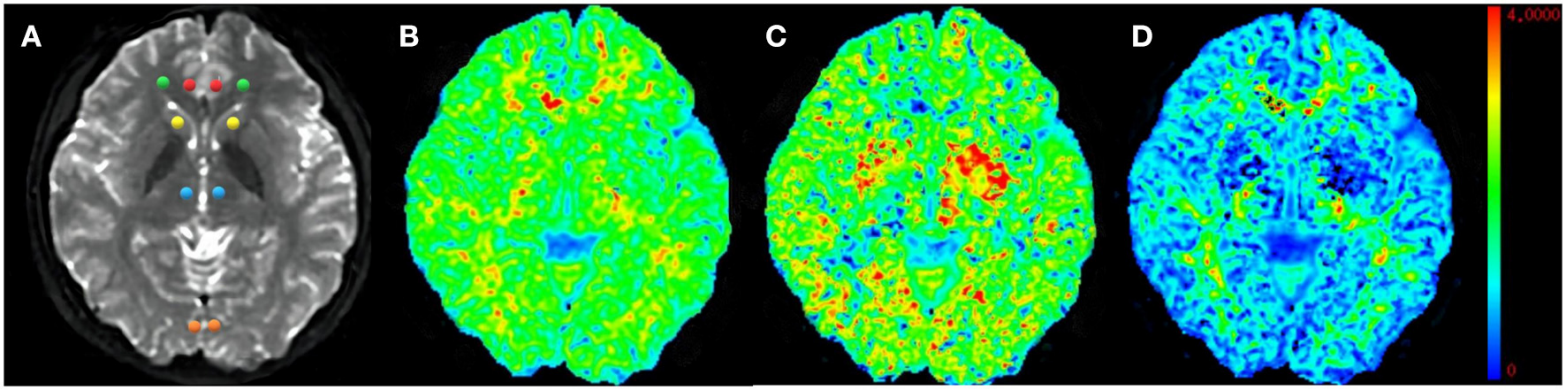
Representative region of interest (ROI) for diffusion kurtosis imaging (DKI) parameters. Raw map of DKI **(A)**, mean kurtosis (MK) map **(B)**, axial kurtosis (Ka) map **(C)**, and radial kurtosis (Kr) map **(D)**. All regions of interests were localized at the axial raw map. The red circle–anterior cingulate cortex (ACC), the orange circle–posterior cingulate cortex (PCC), the green circle–ventral prefrontal white matter (VPWM), the yellow circle–basal ganglia (BG), the blue circle–thalamus (TH).

All DKI data were transferred to a workstation (Advantage Workstation 4.6, GE Medical Systems) using the Functool software package for data processing. DKI metrics, including MK, axial kurtosis (Ka), and radial kurtosis (Kr), were derived using a research tool in the Functool environment developed by GE Applied Science Lab (see http://www.nitrc.org/projects/dke/). DKI software is a research tool in the Functool environment, which was developed by the GE Applied Science Lab. It fifits all DWIs and the minimally-diffusion-weighted image (b0 image) to the DKI model described by the following equation (18).


(1) $$\begin{array}{l} \begin{array}{lll} \ln\;[S(n,b)/S_{0}] & = & -b\sum_{i=1}^{3}\sum_{j=1}^{3}n_{i}n_{j}D_{ij} \end{array}\\ \;\begin{array}{ll} + & \frac{1}{6}b^{2}{\bar{D}}^{2}\sum_{i=1}^{3}\sum_{j=1}^{3}\sum_{k=1}^{3}\sum_{l=1}^{3}n_{i}n_{j}n_{k}n_{l}W_{ijkl} \end{array} \end{array}$$

where S(n, b) is the diffusion signal intensity for diffusion weighting b and diffusion encoding direction n, S_0_ is the signal intensity for b_0_, and D_ij_ and W_ijkl_ are the components of the diffusion and kurtosis tensors, respectively. After the tensors were estimated, the DKI metrics were derived. Eight brain regions, including the ACC, PCC, bilateral ventral prefrontal white matter (VPFWM), bilateral dorsal thalamus (TH), and bilateral basal ganglia (BG), were manually delineated by two experienced radiologists (5–6 years) for ROI analysis (Figure 2) in which each ROI was all approximately 12 mm^2^ in different brain regions and the same size was calculated to minimize the error value. The DKI values for the ROIs were acquired and averaged over three replicates by every radiologist and averaged by two surveyors to correct for inter- and intra-observer errors.

#### 2.3.2. MRS

We initially applied the SAGE software for phase and frequency correction. Metabolite spectral analysis was performed using LCModel (LCModel Inc. Canada). The processing included Fourier transformation and noise filtering, and zero-fill and baseline correction. The metabolite concentrations were then measured. In brief, the original magnetic resonance spectroscopy data were input into the LCmodel software. The software contains a basis set in which there are various metabolite spectral lines of the brain collected under different parameters *in vitro*. These spectral lines contain metabolite concentration and chemical shift information. According to the internal basis set, the software automatically matches and compares with the inputted original spectroscopy line. Finally, the software computes the metabolite concentrations. The features of the LCmodel software is highly automatic, without too much human intervention. Single-voxel spectroscopy was performed on the ACC to obtain a reasonable calibration factor. We applied these data to calibrate metabolite concentrations and calculated the mean absolute concentrations of metabolites such as NAA, MI, Cho, Cr, Glu, and Glx for the eight brain regions, similar to DKI, in all participants.

#### 2.3.3. GluCEST

GluCEST image processing was performed using software routines written in Matlab 7 (Mathworks, Natick, MA, USA). The acquired images were corrected for B_0_ inhomogeneity using a water saturation shift referencing map. The corresponding correction algorithm was discussed in detail by Kim et al. (19). Then, the GluCEST contrast map was generated using the following equation (20):


(2) $$\text{GluCEST=}\frac{\text{S}(\text{−3ppm})\text{ − S}(\text{+3ppm})}{{\text{S}}_{\text{0}}}$$

where S(−3 ppm) and S(+3 ppm) are the images at −3 and +3 ppm, respectively. Z-spectra were obtained from normalized CEST images, and GluCEST% values measured in GluCEST contrast maps were calculated from the same eight brain regions.

### 2.4. Statistical analysis

All statistical analyses were performed using SPSS 20.0 for Windows (IBM, Armonk, NY, USA). For the DKI parameters, brain metabolic data, and GluCEST%, normality and homogeneity were checked, followed by one-way analysis of variance when normality (and homogeneity of variance) assumptions were satisfied. The least significant difference test was used for comparison between groups. Otherwise, the Kruskal–Wallis rank sum test was performed. Pearson′s correlation analysis was used to test the relationships between MRI parameters and clinical values. All measurements are expressed as mean ± standard deviation. A *p* < 0.05 was considered statistically significant.

## 3. Results

### 3.1. Demographic and clinical characteristics

Table 1 shows the demographics and clinical data of all study participants. The disease duration was longer and HDRS scores were higher in patients with BD than in patients with UD. No differences were found across the three groups in terms of education (years), age, or sex ratio (male:female). We conducted correlation analyses between MRS, DKI, GluCEST parameters and disease duration value. The data are displayed in Table 2. However, we observed no correlations in patient with depressed BD and those with UD between MRS, DKI, GluCEST parameters and disease duration value.

**Table 1:** Demographic and clinical information for the participants by group.

**Group**	**Sex (male/female)/*n***	**Age/years**	**Duration of illness/months**	**Education/years**	**HDRS score**
UD (*n* = 30)	13/17	32.2 ± 11.8	48.6 ± 15.1	14.6 ± 3.9	20.7 ± 4.9
BD (*n* = 20)	12/8	34.1 ± 12.4	57.3 ± 14.8	12.3 ± 4.6	24.2 ± 5.4
HC (*n* = 20)	10/10	29.0 ± 8.3	N/A	16.4 ± 4.9	N/A
*p-*value	0.679	0.521	0.041^*^	0.069	0.049^*^

UD, unipolar depression; BD, bipolar disorder; HC, healthy control; HDRS, Hamilton Depression Rating Scale; N/A, not applicable. Data are expressed as mean (standard deviation).

* Statistically significant for BD vs. UD (*p* < 0.05).

**Table 2:** Correlation between disease duration values and MRS, DKI, GluCEST parameters of anterior cingulate cortex and ventral prefrontal white matter.

		**Disease duration values**			
		**UD**		***BD***	
		***R*** **value**	***p-*** **value**	***R*** **value**	***p-*** **value**
Anterior cingulate cortex	MK Glu Cho GluCEST	−0.336 −0.120 0.391 −0.381	0.147 0.645 0.088 0.097	−0.413 0.010 0.190 0.345	0.104 0.976 0.553 0.203
Left ventral prefrontal white matter	MK Glu Cho GluCEST	0.050 0.067 −0.418 0.084	0.155 0.828 0.156 0.739	−0.359 0.026 0.050 0.269	0.841 0.952 0.158 0.353
Right ventral prefrontal white matter	MK Glu Cho GluCEST	−0.020 0.154 −0.241 0.049	0.933 0.583 0.407 0.831	−0.427 0.084 0.231 0.341	0.112 0.844 0.583 0.243

R was a correlation coefficient.

### 3.2. DKI results

**Figure 3:**
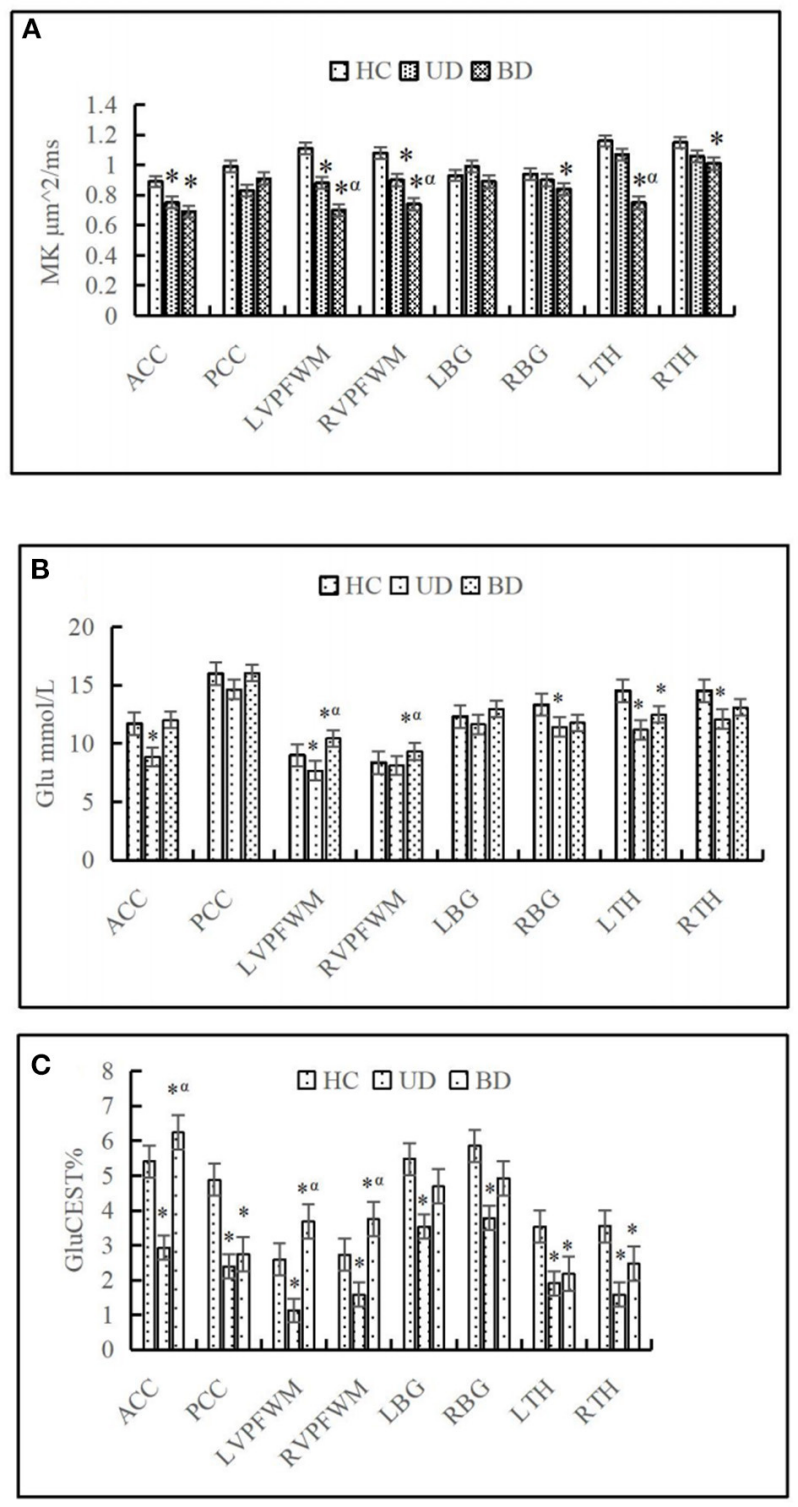
Comparison of mean kurtosis (MK) **(A)**, glutamate (Glu) **(B)**, and chemical exchange saturation transfer (CEST) for glutamate (GluCEST%) **(C)** on eight brain regions in different groups. HC, Healthy control group; UD, unipolar depression group; BD, bipolar disorder group; ACC, anterior cingulate cortex; PCC, posterior cingulate cortex; DPWM, dorsolateral prefrontal white matter; TH, dorsal thalamus; BG, basal ganglia; L, left; R, right. ^*^p < 0.05 was considered to indicate a statistically significant difference compared with the healthy control. ^α^*p* < 0.05 was considered to indicate a statistically significant difference compared with the unipolar depression group.

For the ACC, decreased MK and Kr and increased Ka were found in both the UD and BD groups than the HCs. For the PCC, decreased Kr and increased Ka were found in the UD group than the HCs. For the VPFWM, decreased MK and Kr and increased Ka were found in both the UD and BD groups than the HCs, and MK was reduced more severely in the bilateral VPFWM in the BD group than the UD group. For the BG, increased Ka was found in both the UD and BD groups, and reduced MK was found in the right BG (RBG) in the BD group than the HCs. For the TH, decreased MK was found in the bilateral TH in the BD group than the HCs, and MK was reduced more severely in the left thalamus (LTH) of the BD group than the UD group. These results suggest that compared with HCs, patients with UD and BD showed significantly lower levels of MK and Kr and higher levels of Ka in the brain regions analyzed. Additionally, MK decreased more significantly in the VPFWM and LTH in the BD group. Figure 3A shows MK values in the eight brain regions analyzed in the three groups.

### 3.3. MRS results

A representative MRS spectrum of the ACC obtained using our sequence is shown in Figures 1B–D. Table 3 shows the metabolic values in the eight brain regions analyzed in the three groups. For ACC, compared with the HCs, decreased NAA and MI were found in both the UD and BD groups, decreased Glu was found in the UD group, and higher Cho was found in the BD group than the UD group. For the PCC, no difference in metabolites was found between the UD and HC groups, but increased Cho levels was found in the BD group than the HCs. For the VPFWM, compared with the HCs, decreased NAA and MI were found in the bilateral VPFWM in both the UD and BD groups. Glu levels in the left VPFWM were lower in the UD group but Glu levels in the bilateral VPFWM were higher in the BD group, whereas Cho levels were higher in the BD group. For the BG, compared with the HCs, decreased NAA and MI were found in both the UD and BD groups; a lower Glu in the RBG and higher Glx in the left BG (LBG) were found in the BD group. For the TH, compared with the HCs, decreased NAA and Glx were found in both the UD and BD groups; decreased Glu in the bilateral TH, decreased MI in the LTH and decreased Cho in the RTH were found in the UD group; and decreased Glu in the LTH was observed in the BD group. Figure 3B shows Glu values in the eight brain regions analyzed in the three groups.

**Table 3:** Shows the metabolic values in the eight brain regions analyzed in the three groups.

**Brain region**	**Metabolite (mmol/l)**	**Healthy control**	**Unipolar depression**	**Bipolar disorder**
Anterior cingulate cortex	NAA	10.99 ± 0.15	7.85 ± 0.36^*^	8.37 ± 0.39^*^
	Ins	10.30 ± 0.63	7.38 ± 0.48^*^	7.56 ± 0.74^*^
	Cho	2.95 ± 0.11	3.23 ± 0.35	4.68 ± 0.37^*α^
	Glu	11.69 ± 0.70	8.66 ± 0.47^*^	11.16 ± 0.54^α^
	Glx	13.62 ± 1.04	13.71 ± 0.97	14.26 ± 1.15
Posterior cingulate cortex	NAA	15.46 ± 1.17	12.34 ± 1.13	17.01 ± 2.04
	Ins	7.80 ± 0.66	6.36 ± 0.52	9.45 ± 1.03^α^
	Cho	1.96 ± 0.15	1.46 ± 0.26	2.98 ± 0.68^*α^
	Glu	15.98 ± 0.71	14.26 ± 1.30	17.15 ± 2.01
	Glx	20.48 ± 1.39	18.64 ± 1.04	19.71 ± 2.03
Left ventral prefrontal white matter	NAA	10.05 ± 0.35	7.56 ± 0.47^*^	8.14 ± 0.25^*^
	Ins	8.32 ± 0.68	7.04 ± 0.32^*^	6.94 ± 0.35^*^
	Cho	2.95 ± 0.11	3.07 ± 0.45	4.66 ± 0.27^*α^
	Glu	8.99 ± 0.73	7.16 ± 0.59^*^	10.42 ± 0.54^*α^
	Glx	12.52 ± 1.32	12.15 ± 0.67	13.56 ± 1.03
Right ventral prefrontal white matter	NAA	12.52 ± 0.75	8.97 ± 0.86^*^	10.02 ± 0.23^*^
	Ins	8.17 ± 0.64	6.85 ± 0.30^*^	7.02 ± 0.60^*^
	Cho	2.65 ± 0.20	2.02 ± 0.29	2.39 ± 0.19
	Glu	8.35 ± 0.57	7.47 ± 0.73	9.67 ± 0.36^*α^
	Glx	12.68 ± 1.41	10.68 ± 0.45	14.08 ± 1.25
Left basal ganglia	NAA	12.46 ± 0.56	10.16 ± 0.67^*^	10.35 ± 0.44^*^
	Ins	8.37 ± 0.52	5.70 ± 0.58^*^	6.77 ± 1.05^*^
	Cho	2.65 ± 0.20	2.34 ± 0.26	2.59 ± 0.23
	Glu	12.31 ± 0.92	11.04 ± 0.84	12.68 ± 0.34
	Glx	15.03 ± 1.50	14.96 ± 1.26	17.65 ± 0.64^*^
Right basal ganglia	NAA	14.67 ± 0.43	11.56 ± 0.45^*^	12.03 ± 1.14^*^
	Ins	9.24 ± 0.71	7.15 ± 0.85^*^	7.23 ± 0.92^*^
	Cho	3.85 ± 0.96	2.98 ± 0.74	2.69 ± 0.63
	Glu	13.34 ± 0.77	12.42 ± 1.11	11.02 ± 0.80^*^
	Glx	15.49 ± 0.76	13.96 ± 1.26	16.13 ± 0.49
Left dorsal thalamus	NAA	15.71 ± 1.51	12.09 ± 0.54^*^	12.21 ± 0.47^*^
	Ins	9.74 ± 0.22	7.25 ± 0.41^*^	7.96 ± 0.19
	Cho	3.22 ± 0.50	2.91 ± 0.27	2.64 ± 0.56^*^
	Glu	14.53 ± 0.32	11.21 ± 0.36^*^	12.92 ± 0.68
	Glx	19.49 ± 0.96	15.69 ± 0.65^*^	16.74 ± 0.61^*^
Right dorsal thalamus	NAA	15.45 ± 0.72	12.38 ± 0.47^*^	12.06 ± 0.74^*^
	Ins	9.73 ± 0.86	7.16 ± 0.44^*^	8.85 ± 0.48
	Cho	3.43 ± 0.16	2.36 ± 0.46^*^	2.8 ± 0.45
	Glu	14.68 ± 0.71	11.46 ± 0.58^*^	11.34 ± 0.63^*^
	Glx	18.85 ± 1.03	15.88 ± 1.13^*^	15.06 ± 1.26^*^

* *p* < 0.05 was considered to indicate a statistically significant difference compared with the healthy control.

α *p* < 0.05 was considered to indicate a statistically significant difference compared with the unipolar depression group.

### 3.4. GluCEST results

**Figure 4:**
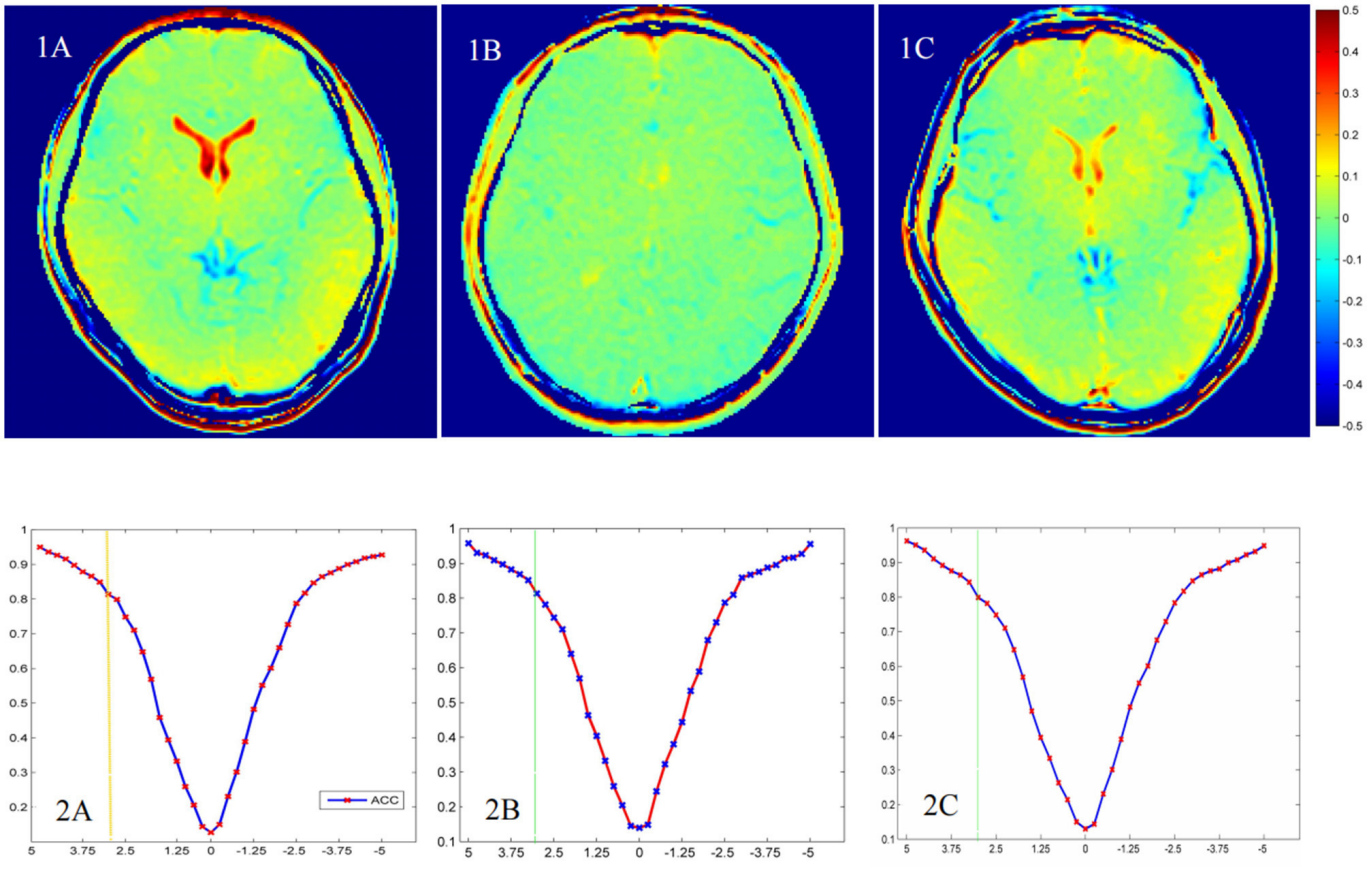
The chemical exchange saturation transfer (CEST) for glutamate (GluCEST) maps **(1A–C)** and z-spectra in the anterior cingulate cortex (ACC) **(2A–C)** of different groups. The healthy control group **(1A, 2A)**, the unipolar depression group **(1B, 2B)**, and the bipolar disorder group **(1C, 2C)**.

Figure 3C shows that the GluCEST% values in most brain regions analyzed were lower in patients with UD than in HCs, whereas those in the ACC and bilateral VPFWM were higher in patients with BD than in HCs or patients with UD. The GluCEST signal intensities in the ACC and bilateral VPFWM were stronger in patients with BD than in HCs and patients with UD, as shown in Figures 4(1A–C). Figures 4(2A–C) shows the z-spectra obtained from HCs, patients with UD, and patients with BD in the ACC. The GluCEST peak was significantly at the 3-ppm line for patients with BD, indicating increased Glu concentrations in patients with BD.

## 4. Discussion

To our knowledge, this is the first study to directly compare differences in the metabolism and microstructural organization of the brain regions in patients with UD, patients with BD, and HCs simultaneously. In this study, the two disorders showed similarities and differences in the metabolic and microstructural changes in the brain regions analyzed, especially the prefrontal brain regions. First, comparisons of the DKI data across the HC, BD, and UD groups revealed brain microstructural alterations in the brain regions analyzed. Compared with HCs, decreased MK, decreased Kr, and increased Ka were noted in both the UD and BD groups, particularly in the ACC and VPFWM, however, decreased MK in the RBG and bilateral TH was only found in the BD group. A lower MK value may suggest a loss of microstructural integrity in the structure (21). Increased Ka may be due to broken axons, increase of basal dendrites, synaptic refinement, and cell density; these processes together with demyelination may result in lower Kr (21). According to the above DKI abnormalities changes, our findings indicated gray and white matter (WM) damage, along with axon and myelin destruction, in a number of brain regions in patients with UD and BP and suggested that brain microstructural abnormalities may overlap in the ACC and VPFWM between these two diseases. In the few studies that assessed BD and UD using DKI, microstructural impairment was noted in the dentate nuclei of the BG (22) and cerebellum (23) in patients with BD, supporting our findings. In addition, Sawamura et al. (10) suggested that decreased MK in the gray matter of the right inferior parietal lobe might have diagnostic value in distinguishing BD from MDD. Although we found decreased MK in the left VPFWM and LTH in patients with BD compared with that in patients with UD, we could not determine whether declined MK in these regions may help in distinguishing BD from UD; thus, a larger sample size is needed for further studies to confirm this.

Based on the advantages of GluCEST, we applied GluCEST combined with multivoxel MRS on 3.0T MRI for patients with BD and UD. The principal findings were as follows: First, the Glu level detected by MRS was significantly higher in the bilateral VPFWM of patients with BD than HCs. Second, patients with BD showed significantly higher Cho levels, whereas patients with UD showed significantly lower Cho levels in the left VPFWM and PCC, compared with HCs. Third, the Glu level detected by GluCEST was lower in more brain regions than that detected by MRS in patients with UD than in HCs, whereas the GluCEST% values in the ACC and bilateral VPFWM were higher in patients with BD than in HCs or patients with UD. Finally, our study proved that GluCEST could be used to provide a new way to detect the changes of glutamate in patients with UD and BD as well as may help in offering a non-invasive neuroimaging measure to distinguish depressed BD from UD.

In line with our study, previous studies have reported attenuated levels of Glu or Glx in patients with UD in the following brain voxels: ACC (24, 25) and medial prefrontal cortex (26). As patients showed statistically significant reductions in MI levels in most regions, alterations in MI levels may reflect glial dysfunction (26). Therefore, abnormalities in glial cell number and function directly affect Glu handling in the brain, reflecting reduced glial cell density, dysfunctional neurotransmission, or downregulation of glutamatergic synapses in the same areas. In addition, our finding is noteworthy because it indicated elevated Glu levels in the DVPFWM and ACC of patients with BD and lower Glu levels in patients with UD. As documented in the majority of ^1^HMRS studies on BD mood episodes (mania, depression), glutamatergic metabolites such as Glx and Glu were higher in multiple brain voxels—such as ACC (13, 27, 28), PCC (29), hippocampus (30), and occipital cortex (31)—in patients with BD than in controls. The results indicate damage to the microstructure detected by DKI, increased cell membrane phospholipid turnover, increased glutamatergic neurotransmission, or excitatory state (32), leading to the accumulation of excessive Glu in BD.

Interesting, when we explored the relationships between MRS, GluCEST and DKI data, we found that both metabolism and microstructure in the ACC and VPFWM changed noticeably in patients with BD compared with other regions evaluated in this study. VPFWM is the major transduction pathway that connects the cortex of the prefrontal lobe and regions of the limbic system. Interestingly, few studies have emphasized on changes in metabolites in the VPFWM of patients with BD. Previous DTI and DKI studies on BD have implicated widespread WM alterations within and beyond the fronto-limbic regions that appear to precede emotional instability (9, 33). Therefore, the VPFWM plays an important role in the pathophysiology of BD. We found that patients with BD had significantly higher Glu and Cho levels in the VPFWM. Given the above findings regarding the change in Glu metabolites, the elevated Cho in patients with BD might be related to MK abnormalities in the integrity of the nerve membrane (33), higher membrane turnover (34), and neurotoxicity due to increased glutamatergic neurotransmission (35). The ACC and VPFWM, which also belong to the prefrontal lobe, play an important role in emotional, motivational, attentional, and executive functions (36). Consequently, Our findings support the significance for the microstructural integrity and brain metabolic changes of the prefrontal lobe region in BD and UD.

The strengths of our study include the use of the newly-implemented combined MRI approach to study microstructure and metabolic differences between patients with UD and BD and the ability of GluCEST to reflect Glu changes and differentiate between these two disorders. However, our study had several limitations. First, the small sample size may have influenced the results. Although we observed metallic and structural alterations in UD and depressed BD, we will increase the sample size to investigate the subgroup (depression, mania, and euthymia) effect in the future. Second, MRS research on metabolite changes during different phases of illness seems indispensable in providing a more detailed insight into the interplay of specific brain metabolites as a putative marker of the longitudinal course of depression (37). We will conduct follow-ups, such as at 6 and 12 months after the first episode of depression, in patients with UD and depressed BD and compare the alterations of MRI parameters to obtain longitudinal microstructural and metabolic alterations in the brain. Third, we are planning to use standardized atlas such as the automated anatomic labeling, for future studies/analyses if possible, which may help support an unbiased measurement. In the future, new prospective studies with larger samples, subgroup research, and regular follow-ups, possibly combining different research modalities, should make it possible to clarify the neurobiological mechanisms of BD and make significant progress in clinical practice.

## 5. Conclusion

Our findings demonstrated that both patients with UD and BD had abnormal microstructural and metabolic alterations, and the changes were not completely consistent in the prefrontal lobe region. Elevated Glu and Cho levels in the ACC and VPFWM of patients with UD and BD may help in differentiating between these two disorders. Our findings support the significance for the microstructural integrity and brain metabolic changes of the prefrontal lobe region in BD and UD.

## Data availability statement

The raw data supporting the conclusions of this article will be made available by the authors, without undue reservation.

## Ethics statement

The studies involving human participants were reviewed and approved by the Second Affiliated Shantou Medical University Hospital and institutional review boards. The patients/participants provided their written informed consent to participate in this study.

## Author contributions

LK, HL, and RW designed this study. HL, FL, WZ, and HZ performed the research and analyzed the data. LK wrote the article. All authors have contributed to the manuscript and approved the submitted version.
